# Resilience-oriented optimization of hospital microgrids with critical load support using ESS and PV under grid outage conditions

**DOI:** 10.1038/s41598-026-34992-x

**Published:** 2026-01-29

**Authors:** Pourya Nazartalab, Hosein Alavi-Rad

**Affiliations:** Department of Electrical Engineering, Lan.C, Islamic Azad University, Langarud, Iran

**Keywords:** Energy storage system, Renewable energy sources, Critical load management, Grid outage modeling, Reliability and resilience indices, Microgrid, Energy science and technology, Engineering

## Abstract

This study develops a resilience-oriented optimization framework for hospital microgrids that integrates photovoltaic (PV) generation, multi-node battery energy storage systems (BESS), and medical load prioritization under grid outage conditions. A mixed-integer linear programming (MILP) model is formulated to jointly optimize ESS scheduling, critical-load support, and renewable utilization across a set of Monte Carlo outage scenarios. The framework introduces a multi-tier hospital load hierarchy (ICU, OR, imaging, pharmacy) based on Value of Lost Load (VOLL), and employs a composite resilience index combining ENS, LOLP, and critical-load survivability. The model is evaluated on modified IEEE 13-, 33-, and 69-bus systems. Results show that coordinated multi-node ESS placement improves resilience significantly, reducing Energy Not Supplied (ENS) by 55–63% compared with baseline configurations, while maintaining ≥ 95% supply to life-critical loads across most stochastic outage realizations. The proposed strategy also ensures stable Resilience Index (RI) values with a variance below 10%, highlighting robustness against PV variability and outage timing uncertainty. Sensitivity analysis demonstrates that ESS capacity, PV penetration, and outage duration are the dominant factors influencing resilience. Overall, the framework provides a practical and quantitatively validated tool for hospital energy planners seeking enhanced survivability and operational security during grid disruptions.

## Introduction

Healthcare facilities require uninterrupted power, yet extreme weather, aging grids, and rising demand threaten reliability. DERs such as PV and batteries now enable microgrids (MGs) and hybrid renewable energy systems (HRES) that support sustainability and critical load recovery. However, despite growing DER deployment, no systematic framework exists to evaluate or optimize resilience across different outage conditions. Most EMS models prioritize cost or efficiency while overlooking outage probability, recovery time, and critical load priorities (e.g., ICU, OR). Additionally, conventional methods ignore stochastic variations in generation and demand, resulting in unrealistic resilience estimates. Thus, a resilience-oriented design and assessment approach is needed.

Energy security is essential for hospitals due to outage risks, and a new framework optimizes on-site storage to improve resilience^1^. While renewable intermittency challenges grid stability, BESS help balance generation–demand and enhance flexibility, with healthcare facilities identified as emerging flexibility providers^2^.

Studies integrating renewables with diesel and storage show improved MG resilience for critical facilities, and the benefits of hospital MGs with PV and batteries have been quantified^3^. Cost-optimal designs for critical-load buildings in the U.S. evaluate lifecycle costs and energy pricing while assessing flexibility from PV, thermal systems, engines, and fuel cells^4^. Hospital-specific storage investment strategies have also been examined due to their high energy consumption^5^, and recent reviews summarize energy storage control methods across scales since 2016^6^.

Hospital MG EMS are key for reliable, cost-effective power, with tailored EMS optimizing storage and usage to prevent power quality failures^7^. Increasing renewable energy, including solar water heaters, supports sustainability^8^. Fuel cell combined heat and power (FC-CHP) with solar PV improves hospital efficiency^9^.

Hybrid renewables serve off-grid rural hospitals with power and medical oxygen^10^. Powering biomedical devices remains challenging, driving demand for energy-harvesting solutions^11^. Renewable applications in medical/dental fields and hospital heating/cooling have been reviewed^12,13^. Controlled environments for transplant care require minimizing energy-related pollution^14^.

Miniaturized wearable medical devices increase demand for compact, high-capacity batteries, reducing reliance on nonrenewables^15^. Wind power variability challenges grid stability, addressed by hybrid ultra-capacitor storage and fuzzy pitch control in a 50 kW turbine^16^. Expanding MGs require flexible power management; a cost- or demand-based scheduling system using Python and databases has been developed^17^. Improved PV MG sizing with hybrid battery-supercapacitor storage uses MILP and battery replacement planning^18^. A hybrid renewable system with hydrogen storage has been modeled for hospital wards treating COVID-19 patients^19^.

Electrical MGs are small-scale power systems with localized generation and limited loads. A hybrid renewable MG for a rural area, including PV, wind, biomass, batteries, diesel, and EVs, was optimized in^20^. Extreme weather causes major outages; standby redundancy with dual energy setups was proposed to support critical loads during blackouts^21^. Renewable-based MGs for disaster resilience in healthcare were developed^22^, and grid resilience was improved via diesel-grid or grid-connected PV-BESS MGs^23^. A distributed heuristic algorithm for critical load restoration enhances reliability and efficiency by islanding controllable generators^24^. Autonomous islanded MG operation for post-outage restoration has also been emphasized^25^.

The shift toward nearly zero-energy buildings (NZEBs) using hybrid MGs with cooling, heating, and DERs, including hybrid storage, has been studied with emphasis on critical loads^26^. Home-integrated MG planning and MG-based critical load restoration frameworks have also been explored^27,28^. Increasing wind energy reliance raises cybersecurity risks like false data injection attacks, which can disrupt power output significantly^29^. Optimal hybrid MG designs combining solar, wind, batteries, and diesel generators have been proposed^30^. Hospital energy management, especially during COVID-19, focusing on integrated power and thermal systems, has been analyzed^31^.

Recent focus on MG EMS includes a new classification of EMS approaches^32^. An EMS framework optimizing battery charge cycles to reduce costs under dynamic tariffs and variable renewables was developed for grid-connected MGs^33^. Ensuring resilience for critical infrastructures (CIs) like hospitals involves categorizing CIs by service domain and emphasizing power supply to CLs during disruptions^34^. System-level planning and restoration strategies for CLs have been addressed^35^. Mechanical reliability improvements include turbine blade damage detection via frequency analysis for predictive maintenance^36^. In biomedical fields, battery-free powering of implantable and wearable devices through advanced energy harvesting and conditioning methods has been reviewed^37^, with triboelectric nanogenerators demonstrated for self-powered health monitoring^38^.

Table 1 provides a concise summary of representative prior studies, outlining their main objectives, modeling approaches, and the evaluation metrics commonly used, such as ENS, reliability indices, and resilience indicators. This comparison highlights that existing works rarely incorporate hospital-specific load prioritization, stochastic outage modeling, or coordinated multi-node ESS-PV optimization, underscoring the need for the integrated framework proposed in this study.

**Table 1:** Summary of key previous studies and their evaluation metrics.

References	Focus Area	Methods/key features	Evaluation metrics
^1^	ESS for mission-critical facilities	Resilience-oriented ESS operation	ENS, Resilience Score
^3^	Hospital MG (PV + ESS)	MG optimization under outages	ENS, Autonomy
^18^	Healthcare PV, storage MG	MILP-based optimal design	ENS, Reliability
^23^	Hospital MG vs. diesel backup	Comparative resilience modeling	ENS, Resilience Index
^28^	Critical-load restoration	Network reconfiguration	ENS, Restoration Time

The primary objective of this study is to develop and validate a resilience-centric framework for the operation and planning of hospital-based MGs using DERs and BESS. Specifically, the paper aims to:

- Formulate a MILP model to prioritize critical loads and optimize energy storage dispatch.
- Quantify performance metrics including Loss of Load Probability (LOLP), Energy Not Supplied (ENS), and a Resilience Index under varying outage durations.
- Assess system behavior under uncertainty using Monte Carlo simulations on IEEE 13-, 33-, and 69-bus test systems.
- Perform sensitivity analyses on key parameters: ESS capacity, PV output, outage duration, and Value of Lost Load (VOLL).

Unlike prior studies which primarily focus on economic optimization or renewable integration alone, this paper introduces a multi-layered methodology that jointly evaluates technical reliability, operational resilience, and critical load recovery under diverse and realistic conditions.

This study provides several contributions that distinguish it from prior work:

- *Integrated Resilience-Oriented Framework* We develop a unified nine-step framework that combines stochastic outage-driven scenario generation, priority-based load classification using VOLL, and a resilience-driven MILP optimization model. While individual components exist in the literature, their integration for resilience assessment of healthcare MGs has not been previously demonstrated.
- *Hospital-Centric Operational Modeling* The model explicitly incorporates medical-load criticality, outage survivability requirements, and ESS–PV coordination customized for hospital operational constraints—addressing a gap where most existing studies treat loads uniformly.
- *Resilience Metrics Under Realistic Disturbances* The framework evaluates ENS, LOLP, and composite resilience indicators across a large set of stochastic outage scenarios, offering a more realistic and quantitative assessment than deterministic or single-scenario analyses commonly used in earlier works.
- *Comprehensive Sensitivity Analysis* By varying ESS size, outage duration, PV uncertainty, and VOLL, the study identifies the conditions under which hospital resilience can be significantly improved—providing actionable insights for planners and operators.

These contributions collectively advance the state of the art by demonstrating a coherent and practically relevant methodology specifically tailored to the resilience needs of hospital MGs.

This work directly contributes to resilient healthcare infrastructure by providing actionable guidelines for system planners, hospital engineers, and energy policymakers.

The novelty of this study lies in the development of a unified resilience-oriented optimization framework specifically tailored to hospital MGs. Unlike existing works that treat critical loads uniformly or rely on deterministic outage assumptions, the proposed method introduces: (i) a multi-tier medical load prioritization scheme based on VOLL that differentiates ICU, OR, imaging, and pharmacy loads; (ii) a stochastic outage modeling process using Monte Carlo simulation to capture realistic uncertainty in outage timing, PV variability, and load demand; and (iii) coordinated multi-node ESS scheduling embedded in a resilience-focused MILP formulation. This integrated approach enables simultaneous evaluation of ENS, LOLP, and a composite resilience index, providing a more comprehensive and clinically meaningful assessment of hospital survivability under disruptive grid conditions.

The paper systematically develops a hospital MG resilience framework, beginning with an introduction to system components, load classification, and resource modeling ( “System modeling and theoretical background” section). It then outlines the methodology, including MILP formulation and resilience assessment (“Methodology” section). Simulation results on IEEE test systems under outage scenarios are presented in “Case studies and results” section, followed by sensitivity analyses of key design parameters in “Sensitivity analysis” section. The findings are discussed in “Discussion” section, and the paper concludes with key insights and future research directions in “Conclusion” section.

##  System modeling and theoretical background

This section describes the hospital MG modeling framework, including test system setups, component modeling (PV, BESS, load decomposition), and the scenario framework for performance evaluation under different conditions.

### Test systems configuration

Standard IEEE 13-, 33-, and 69-bus networks are used to represent MG setups with various scales. Each is configured to include DERs and critical hospital loads^17^.

#### 13-Bus, 33-Bus, and 69-Bus MG architectures

The IEEE 13-, 33-, and 69-bus radial distribution networks are employed to simulate MG scenarios. These systems are adapted to include^17–19^:

- DERs, such as PV systems and BESS.
- Healthcare-critical infrastructure (modeled as priority loads).
- The capability to transition between grid-connected and islanded modes.

Each system is represented as a graph G=(N, L), where:

- N: set of buses.
- L: set of distribution lines with impedance Z_ij_=R_ij_+jX_ij_.

Bus voltages V_i_, power injections S_i_=P_i_+jQ_i_, and line currents I_ij_ are modeled using a forward/backward sweep algorithm suitable for radial topologies. The admittance matrix Y_bus_ is used in standard load flow analysis.

#### Critical load placement strategy

Critical healthcare loads—including ICUs, ORs, imaging suites, and pharmacies—are strategically mapped onto buses with high reliability and DER support.

Let N_C_⊂N denote buses with critical medical infrastructure. Selection criteria include^20–28^:

- Electrical proximity to DERs.
- Voltage stability.
- Feeder reliability.
- Load density.

To accurately represent hospital operation, the load model is expanded beyond a generic “critical load” definition. Hospital demand is categorized into three medical-specific groups:

(i) Life-support and emergency services (e.g., ICU, OR, ER equipment), which must be fully supplied at all times;
(ii) Clinical and diagnostic loads (laboratories, imaging, sterilization), which require high but non-absolute supply continuity; and.
(iii) Supportive facility loads (HVAC, lighting, administration), which are partially curtailable.

Each category is assigned a distinct VOLL, minimum service level, and curtailment constraint that directly affects the MILP decisions and scenario outcomes. This hospital-oriented structure ensures that the optimization reflects real operational priorities rather than treating all critical loads uniformly.

### Component models

This section models PV generation, battery storage dynamics, and decomposed hospital loads to capture operational variability and priorities.

#### PV generation profiles

PV systems are modeled as time-varying, weather-dependent generation units. The power output P_PV, i_(t) at bus i is given by^10^:

1 $${P_{PV,i}}\left( t \right)={\eta _i}{A_i}{G_t}\left( t \right)$$

where, η_i_ is efficiency of PV array, A_i_ is surface area of PV panels at bus i, and G_t_(t) is solar irradiance at time t.

Generation profiles are derived from irradiance datasets (e.g., NREL Typical Meteorological Year, TMY), scaled for regional conditions and system capacity.

So, RES penetration is calculated as^8^:

2 $$RES{\text{ }}Penetration\left( \% \right)=\frac{{{P_{PV}}}}{{{P_{Load}}}} \times 100$$

#### Battery energy storage system (BESS) dynamics

Each BESS is modeled with:

- Charge/discharge power: P_i_^ch^(t), P_i_^dis^(t).
- State-of-charge (SOC): E_i_(t).
- Efficiency: η_c_,η_d_.

Energy balance is expressed as^7^:

3 $${E_i}\left( {t+1} \right)={E_i}\left( t \right)+{\eta _c}P_{i}^{{ch}}\left( t \right)\Delta t - \frac{{P_{i}^{{dis}}\left( t \right)}}{{{\eta _d}}}\Delta t$$

Subject to:

4 $$E_{i}^{{\hbox{min} }} \leqslant {E_i}\left( t \right) \leqslant E_{i}^{{\hbox{max} }}{\text{ }},{\text{ }}0 \leqslant P_{i}^{{ch}}{\text{ }},{\text{ }}P_{i}^{{dis}} \leqslant P_{i}^{{rated}}$$

The BESS is coordinated to support:

- Critical load supply during outages.
- Grid services during normal operation.
- PV smoothing or arbitrage.

#### Hospital load decomposition

Hospital loads are divided into operational segments with specific priorities, load profiles, and curtailment tolerances (Table 2). This allows detailed resilience analysis and tailored energy supply strategies based on each subsystem’s criticality^37,38^.

**Table 2:** Categorization of hospital sub-loads based on criticality, operational profile, and curtailability.

Load type	Typical bus location	Criticality	Load type	Curtailability
ICU	Near BESS	High	Constant	No
OR	Medium voltage buses	High	Peaky	Limited
Imaging	Near PV + Storage	Medium	Intermittent	Partial
Pharmacy	General load buses	Medium	Stable	Partial

Let Each sub-load is modeled with either static or time-varying demand curves, using known hospital load data or synthetic profiles.

### Scenario framework

Normal and contingency conditions are modeled, including outage durations and load prioritization, to evaluate system resilience.

#### Base case Vs. contingency scenarios

The system is evaluated under two scenario classes:

- Base Case: All components operational; grid support available.
- Contingency Scenarios:
- Grid outage (partial/full).
- DER or line failure (N-1, N-2).
- Peak load stress with PV variability.

The contingency state at time t is denoted C(t)∈{0,1,…}, where 0 represents nominal operation. The supply-demand balance constraint under contingency is:

5 $$P_{i}^{{DER}}\left( t \right)+P_{i}^{{BESS}}\left( t \right) \geqslant P_{i}^{{crit}}\left( t \right){\text{ }}\forall i \in {{\mathbb{N}}_c},{\mathbb{C}}\left( t \right)>0$$

#### Outage duration modeling

To reflect realistic disruption durations, outage windows are modeled stochastically or deterministically:

- Fixed Duration Model: Predefined outage interval [t_s_,t_e_].
- Stochastic Duration: Outage time T_out_∼Exp(λ).

Let δ_i_(t) = 1 if grid supply at bus i is disrupted at time t, and 0 otherwise.

Load curtailment for bus i is^30–33^:

6 $$L{C_i}\left( t \right)=\hbox{max} \left( {0,P_{i}^{{load}}\left( t \right) - P_{i}^{{DER}}\left( t \right) - P_{i}^{{BESS}}\left( t \right)} \right).{\delta _i}\left( t \right)$$

Metrics such as ENS and LOLE are computed over the outage window.

## Methodology

This section outlines the methodology for evaluating MG performance during contingencies, emphasizing critical load support in hospitals. It includes a constrained optimization model, reliability metrics, and the simulation process.

### Optimization problem formulation

The MG operation is modeled as a MILP problem to optimize critical load supply during islanded mode, balancing generation, storage, and demand while minimizing outage impacts.

#### Objective function

The objective is to maximize critical load supply during grid outages over 24 h, expressed mathematically as:

7 $$\hbox{max} \sum\limits_{{t=1}}^{T} {\sum\limits_{{i=1}}^{{{N_L}}} {{\omega _i} \cdot P_{{i,t}}^{{served}}} }$$

Where, P_i, t_^served^ is the power supplied to load i at time t, ω_i_ is the criticality weight assigned to load i (e.g., higher for ICU or OR), T is the number of time steps (typically T = 24), and N_L_ is the total number of loads.

Alternative objective functions may incorporate minimizing unmet energy or maximizing resilience metrics.

####  Constraints

The optimization is subject to several physical and operational constraints:

i. Power Balance Constraint:

For each time step t, power generation, storage discharge, and load must be balanced:

8 $$\sum\limits_{{j=1}}^{{{N_G}}} {P_{{j,t}}^{{gen}}+P_{t}^{{dis}}} =\sum\limits_{{i=1}}^{{{N_L}}} {P_{{i,t}}^{{served}}+P_{t}^{{ch}}}$$

where, P_j, t_^gen^ is the power from generation source j, P_t_^dis^ and P_t_^ch^ are battery discharging and charging powers.

ii. SOC Limits:

9 $$SO{C_{\hbox{min} }} \leqslant SO{C_t} \leqslant SO{C_{\hbox{max} }}$$

SOC dynamics:

10 $$SO{C_{t+1}}=SO{C_t}+{\eta _{ch}}.P_{t}^{{ch}}.\Delta t - \frac{{P_{t}^{{dis}}.\Delta t}}{{{\eta _{dis}}}}$$

Where, η_ch_, η_dis_ are charging/discharging efficiencies, and Δt is the time step duration (e.g., 1 h).

iii. PV Curtailment:

PV generation can be curtailed based on operational constraints or load demand:

11 $$0 \leqslant P_{t}^{{PV - used}} \leqslant P_{t}^{{PV - avail}}$$

with curtailed energy:

12 $$P_{t}^{{PV - curt}}=P_{t}^{{PV - avail}} - P_{t}^{{PV - used}}$$

### Reliability metrics

To assess MG performance under outage scenarios, we use the following metrics.

#### LOLP

This index quantifies the probability that available generation and storage are insufficient to meet critical demand:

13 $$LOLP=\frac{{\sum\nolimits_{{s=1}}^{S} {{\delta _s}} }}{S}$$

where, δs = 1 if load shedding occurred in scenario s, and 0 otherwise, and S is the total number of Monte Carlo scenarios.

LOLP is used as the main reliability index because it measures the probability that the MG cannot meet critical hospital loads under uncertainties in renewable output, storage behavior, and load variations. This aligns with the study’s focus on stochastic outage conditions. In contrast, Line Outage Distribution Factor (LODF) is designed for deterministic N-1 transmission line contingency analysis and does not match the stochastic reliability objectives of the proposed framework. Therefore, LOLP is the more suitable metric for evaluating hospital MG resilience.

#### ENS

ENS measures the total unsupplied energy across all time steps and load points:

14 $$ENS=\sum\limits_{{t=1}}^{T} {\sum\limits_{{i=1}}^{{{N_L}}} {\left( {P_{{i,t}}^{{load}} - P_{{i,t}}^{{served}}} \right) \cdot\Delta t} }$$

It reflects both frequency and severity of outages.

#### Resilience index

Resilience index measures how well the system maintains critical load supply during disruptions:

15 $$RI=\frac{{\sum\nolimits_{{t=1}}^{T} {\sum\nolimits_{{i=1}}^{{{N_L}}} {{\omega _i} \cdot P_{{i,t}}^{{served}}} } }}{{\sum\nolimits_{{t=1}}^{T} {\sum\nolimits_{{i=1}}^{{{N_L}}} {{\omega _i}\cdot P_{{i,t}}^{{load}}} } }}$$

A higher RI indicates more resilient performance.

### Simulation workflow

To capture the stochastic nature of outage events and PV generation, the simulation procedure follows this framework.

#### 24-Hour time horizon

The optimization is executed over a 24-hour window with 1-hour resolution. Load and generation profiles are modeled accordingly.

#### Monte Carlo for outage events

Multiple Monte Carlo scenarios are generated to simulate random outage start times and durations. For each scenario:

- A random outage window is defined.
- The optimization problem is solved.
- Reliability metrics are computed.
- Results are averaged across all scenarios.

Each scenario captures variability in PV output, load fluctuations, and storage response, ensuring a statistically robust assessment of system performance.

### Methodology process

**Fig. 1:**
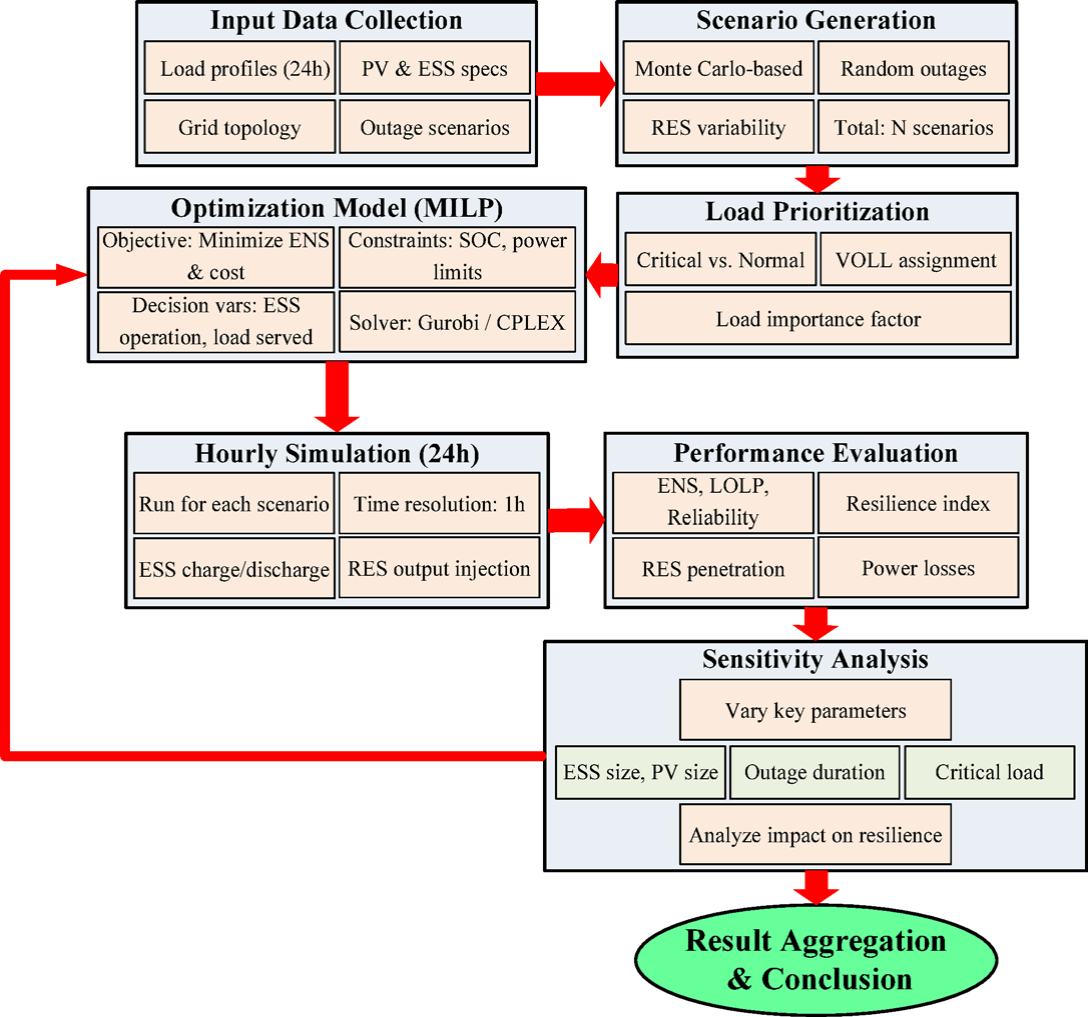
Methodology process.

The proposed methodology follows a structured nine-step framework (Fig. 1) to evaluate the resilience and operational performance of distribution networks with ESS and PV under outage conditions:

- *Input Data Collection* Load profiles, DER specifications, outage parameters, and network data for the IEEE 33- and 69-bus systems are compiled and pre-processed.
- *Scenario Generation* Monte Carlo simulations generate stochastic scenarios capturing variations in PV output, load uncertainty, and outage timing/duration.
- *Load Prioritization* Loads are classified as critical or non-critical based on the Value of Lost Load (VOLL), guiding supply decisions during constrained conditions.
- *MILP Optimization Model* A mixed-integer linear programming model minimizes ENS and operational cost while determining optimal ESS scheduling under power balance, SOC, and network constraints.
- *Hourly Simulation* A 24-hour time horizon is simulated hour-by-hour to capture ESS charging/discharging patterns, load supply levels, renewable contributions, and system losses.
- *Performance Evaluation* For each scenario, resilience and reliability indicators—including LOLP, ENS, and composite resilience indices—are computed.
- *Sensitivity Analysis* Key parameters (ESS capacity, outage duration, PV production variability, and VOLL) are varied to assess their impacts on resilience outcomes.
- *Result Aggregation* Outputs from all scenarios are aggregated into tables and graphical representations showing SOC trajectories, load supply proportions, and ESS operational behavior.
- *Conclusion and Insight Extraction* The final step synthesizes results to identify effective ESS control strategies, resilience limitations, and planning recommendations for distribution networks.

## Case studies and results

This section evaluates hospital MG performance on IEEE 13-, 33-, and 69-bus systems with PV, BESS, and prioritized critical loads (ICU, OR, Imaging, Pharmacy). Outage scenarios test resilience under uncertainty. The 13-bus feeder represents a compact MG with four buses serving critical hospital functions.

- Bus 7: ICU
- Bus 8: OR
- Bus 9: Imaging
- Bus 11: Pharmacy

Three PV units (70 kW, 120 kW, 60 kW) are installed at buses 3, 9, and 12. BESS units at buses 4, 7, and 10 provide 1.8 MWh capacity and 650 kW power, with 50% initial SOC. See Table 3 for details.

**Fig. 2:**
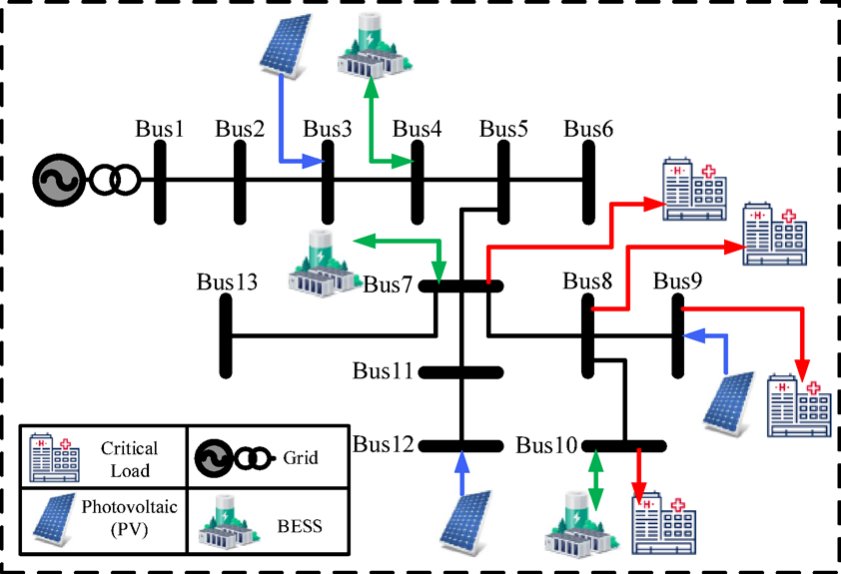
IEEE 13-bus test system.

Four outage scenarios—from minor faults to rolling blackouts—are modeled. Figure 2 shows the network topology and placement of PVs, BESSs, and critical loads. The IEEE 33-bus feeder represents a medium-scale hospital or district medical center, with four critical buses selected for essential services.

- Bus 6: ICU
- Bus 11: OR
- Bus 20: Imaging
- Bus 29: Pharmacy

Four PV units (60–120 kW) are installed at buses 4, 10, 21, and 29. BESS units at buses 14, 18, 22, and 25 provide 2.4 MWh energy and 900 kW power, with SOC constraints prioritizing critical loads. Details are in Table 4.

**Table 3:** IEEE 13-bus system specifications.

Parameter	Value
Critical Buses	7 (ICU), 8 (OR), 9 (Imaging), 11 (Pharmacy)
PV buses & capacities (kW)	3 (70), 9 (120), 12 (60)
BESS buses	4, 7, 10
BESS capacities (kWh)	500, 800, 500
BESS power ratings (kW)	200, 250, 200
Initial SOC (%)	50% for all units
SOC limits (%)	≥ 30% (critical), ≥ 20% (normal loads)
Outage scenarios	Minor, medium, major, rolling blackout

**Table 4:** IEEE 33-bus system specifications.

Parameter	Value
Critical buses	6 (ICU), 11 (OR), 20 (Imaging), 29 (Pharmacy)
PV buses & capacities (kW)	4 (70), 10 (120), 21 (60), 29 (80)
BESS buses	14, 18, 22, 25
BESS capacities (kWh)	500, 800, 500, 600
BESS power ratings (kW)	200, 250, 200, 250
Initial SOC (%)	50%
SOC limits (%)	≥ 30% (critical), ≥ 20% (normal loads)
Outage scenarios	Identical to IEEE 13-bus system

**Fig. 3:**
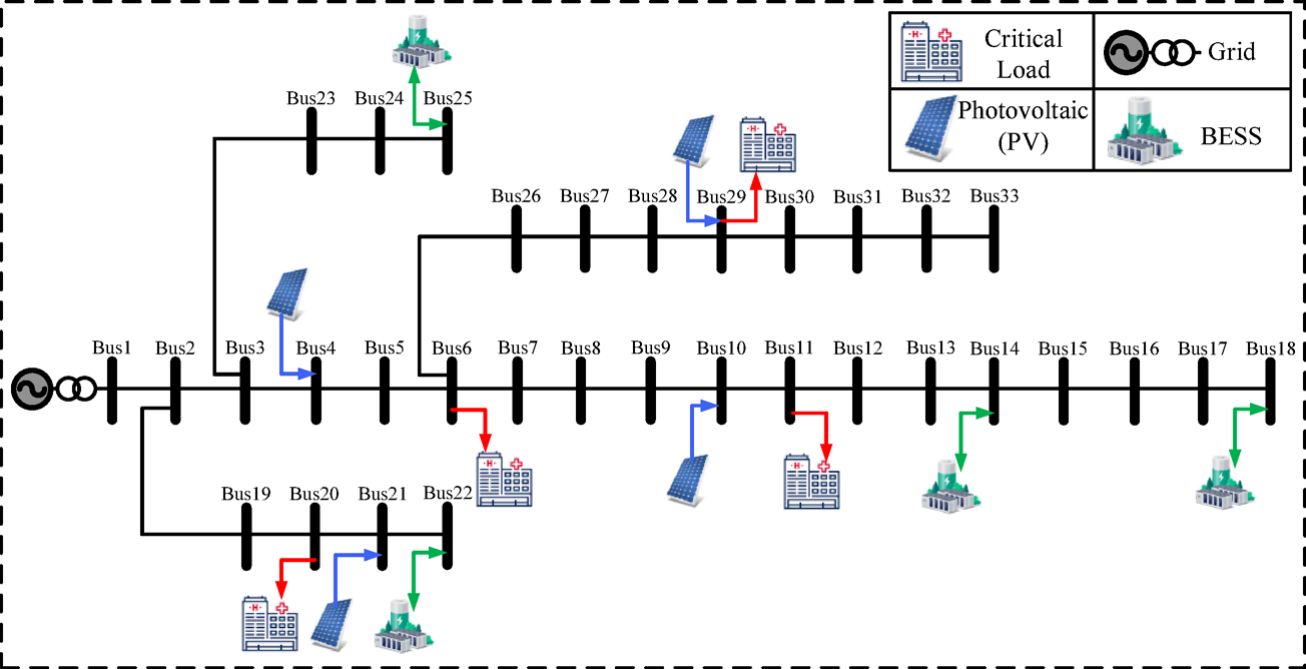
IEEE 33-bus test system.

Figure 3 shows the network, including PV, BESS, and critical load locations. The IEEE 69-bus feeder represents a large-scale hospital grid with expanded PV and storage, assigning critical services to:

- Bus 15: ICU
- Bus 32: OR
- Bus 48: Imaging
- Bus 63: Pharmacy

Eight PV units (20–60 kW) are installed at buses 9, 17, 25, 34, 41, 49, 57, and 66. Six BESS units at buses 11, 20, 33, 39, 50, and 59 provide 3.6 MWh storage and 1.35 MW power. Full specs are in Table 5.

**Table 5:** IEEE 69-bus system specifications.

Parameter	Value
Critical buses	15 (ICU), 32 (OR), 48 (Imaging), 63 (Pharmacy)
PV buses & capacities (kW)	9 (50), 17 (20), 25 (35), 34 (20), 41 (60), 49 (50), 57 (20), 66 (40)
BESS buses	11, 20, 33, 39, 50, 59
BESS capacities (kWh)	500, 800, 600, 500, 600, 600
BESS power ratings (kW)	200, 250, 250, 200, 200, 250
Initial SOC (%)	50%
SOC limits (%)	≥ 30% (critical), ≥ 20% (normal loads)
Outage scenarios	Consistent with previous systems

**Fig. 4:**
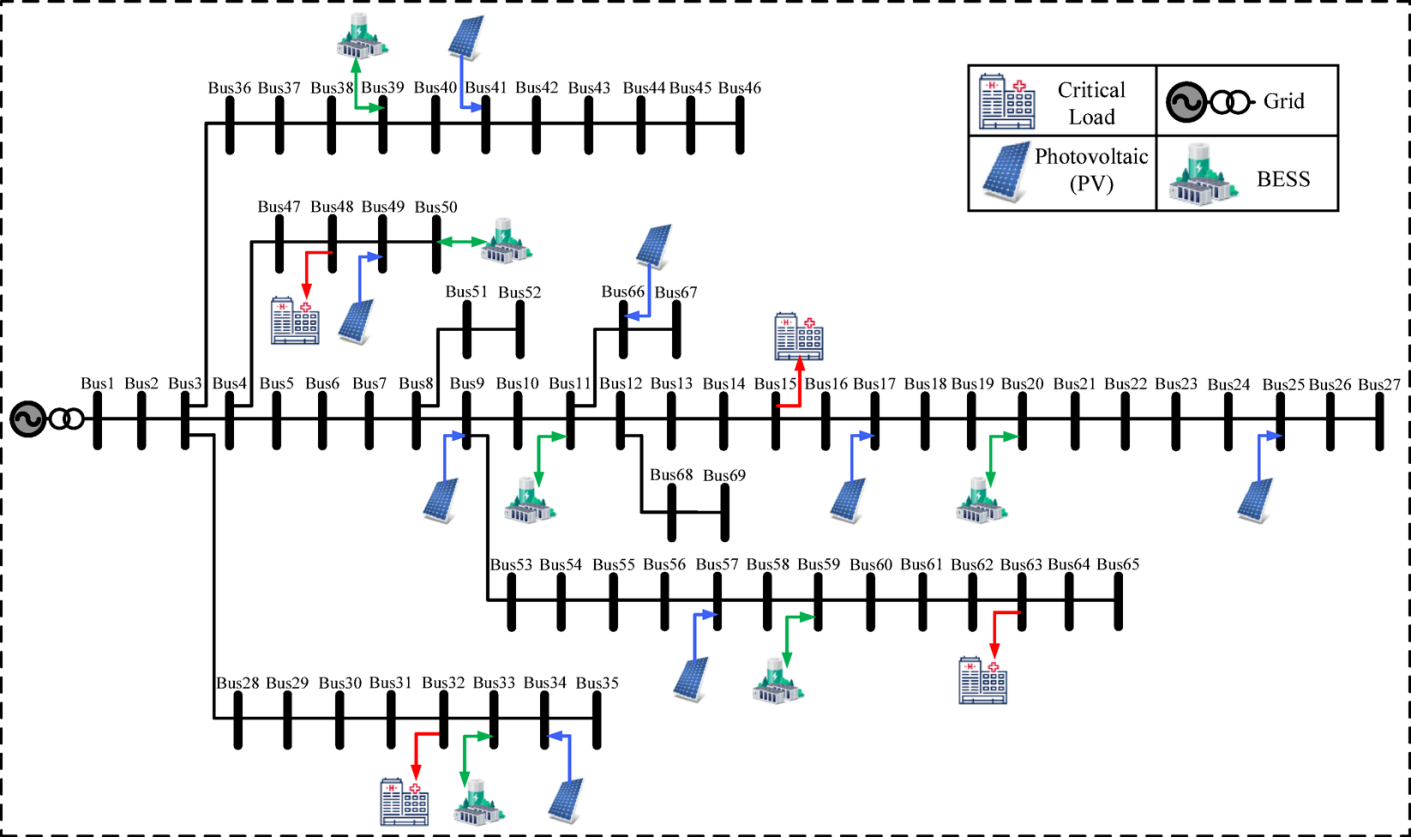
IEEE 69-bus test system.

The layout of PVs, BESSs and critical loads are shown in Fig. 4, reflecting distributed architecture and enhanced resilience potential. Figures 2, 3 and 4 were created using Microsoft Visio.

### Results of IEEE 13-Bus test system

A 24-hour simulation on the IEEE 13-bus network evaluated the impact of distributed ESS and RES during outages. ESS units at buses 4, 7, and 10 had capacities of 500–800 kWh and power ratings of 200–250 kW. Performance metrics included power losses, renewable penetration, load continuity, and resilience.

#### Grid losses and renewable integration trends

**Fig. 5:**
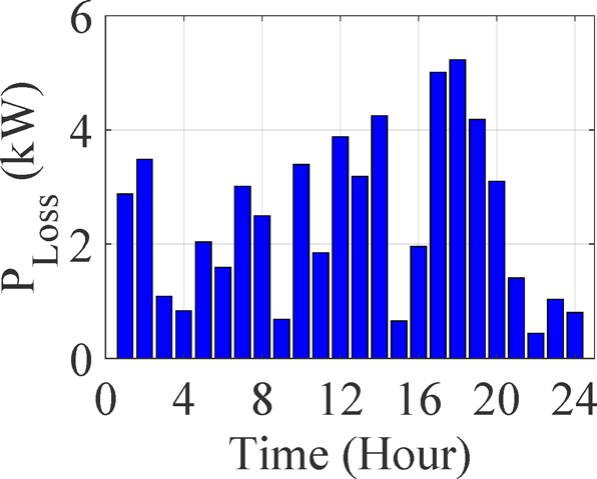
Active power loss of 13-bus test system in 24-hours.

**Fig. 6:**
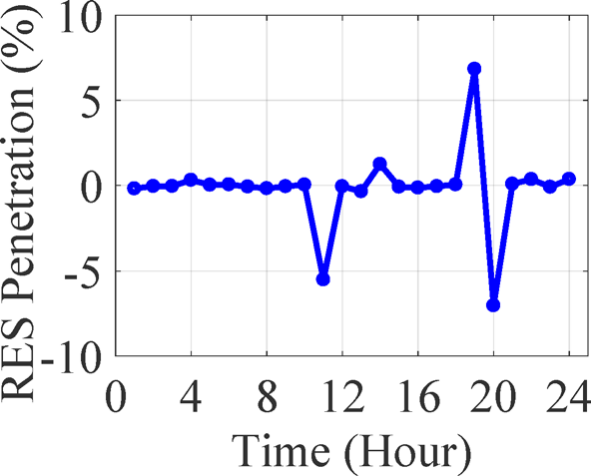
RES penetration of 13-bus test system in 24-hours.

Figure 5 shows active power losses over 24 h, and Fig. 6 displays hourly renewable energy penetration.

Overall, the system exhibits dynamic fluctuations driven by diurnal solar profiles and load variations.

- Power losses vary from under 1 kW to over 1000 kW, closely tracking moments of heavy discharging and network reconfiguration due to outages.
- RES penetration shows an erratic pattern, with both exceptionally high and negative values observed. For instance, penetration exceeding 1000% in some hours suggests surplus generation feeding into the grid or being curtailed due to limited demand or storage space.
- Conversely, negative RES penetration values likely stem from modeling artifacts where RES output is treated as a deficit, possibly due to net metering imbalance, or as a result of negative demand nodes (e.g., due to reverse flows from DERs). These anomalies highlight the need for robust normalization strategies when computing relative RES contribution.

This behavior underscores the challenge of integrating variable RES in a constrained network without adequate real-time balancing via ESS.

This is further quantified in Table 6, showing the standard deviation in power losses reaching over 200 kW. This table summarizes the aggregated metrics of the 13-bus system, highlighting the wide variability in RES penetration and load supply conditions. Notably, reliability and resilience values fluctuate due to both stochastic RES behavior and outage scenarios.

**Table 6:** Aggregated performance metrics (24-hour overview of IEEE 13-bus system).

Metric	Min value	Max value	Mean value	Std. dev
System power loss (kW)	0.57	1031.79	127.96	213.44
RES penetration (%)	− 7024.2	6837.0	− 412.8	1493.27
Battery utilization (%)	16.4	74.7	38.9	14.65
Critical load supplied (%)	− 477.4	192.2	− 7.6	116.83
LOLP	-87.725	114.682	1.87	21.45
ENS (kWh)	0.0	572391.1	173221.6	182306.4
Reliability index	− 113.682	88.725	0.13	28.53
Resilience index	− 4.774	1.310	0.112	1.24

#### Load supply reliability and energy not served

**Fig. 7:**
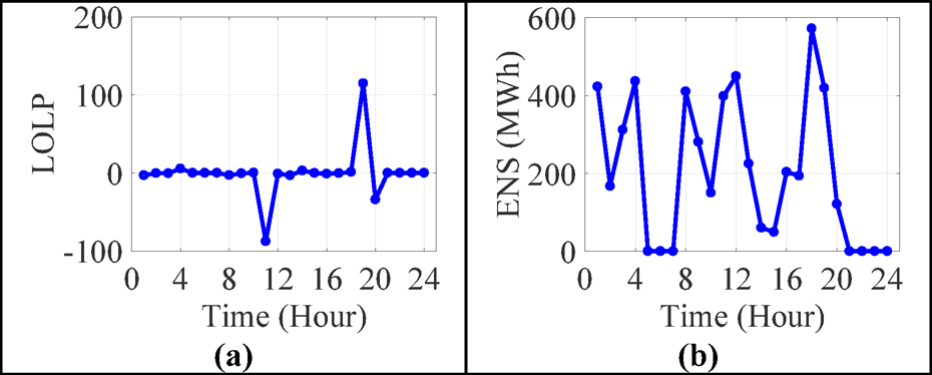
Load results of 13-bus test system in 24-hours (**a**) loss of load probability (**b**) energy not supplied.

Figure 7a and b capture the LOLP and ENS, two critical indices for reliability assessment:

- In several intervals, LOLP values fall below zero, which is physically invalid, since LOLP represents a probability and must be ≥ 0. These deviations likely originate from incorrectly computed available capacity or misinterpreted surplus conditions (e.g., when RES generation exceeds total demand).
- Despite these computational outliers, general ENS values trend logically, with higher values appearing during peak load hours and lower when ESS discharging is effectively coordinated.

From a reliability planning perspective, this suggests the ESS dispatch logic is reasonably effective in meeting demand, albeit with room for refinement in abnormal event handling (such as high RES surpluses or ESS saturation).

#### ESS dynamics and load resilience

**Fig. 8:**
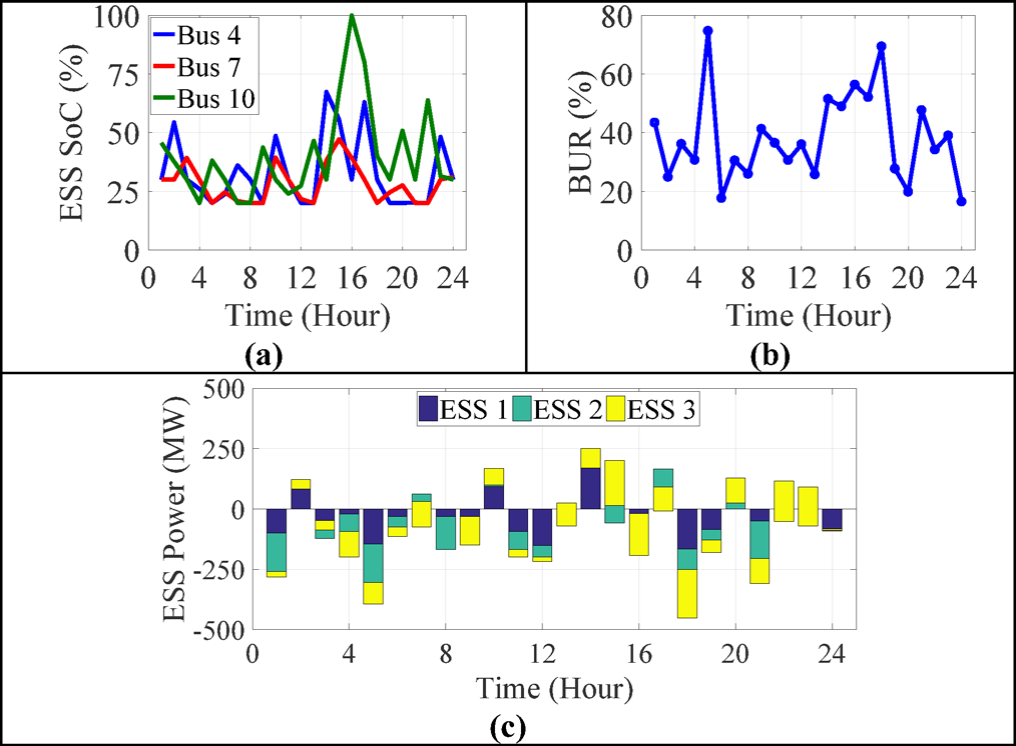
ESS results of 13-bus test system in 24-hours (**a**) state of charge (**b**) battery utilization ratio (**c**) power allocation.

Figure 8 disaggregates ESS performance into three layers: SOC, BUR, and Power Allocation.

- SOC trajectories display expected charge-discharge patterns, with SOC dipping in the early hours and partially recovering through mid-day charging cycles.
- Battery Utilization peaks when the system experiences simultaneous RES curtailment and high demand, validating the role of ESS as a buffer.
- Power allocation across the three storage units reflects a heterogeneous control logic, where ESS at different buses respond asynchronously to load or RES conditions, supporting the grid in a decentralized fashion.

Table 7 summarizes that ESS units at Buses 4 and 10 underwent more frequent and deeper cycling, reflecting their proximity to load centers. All units operated within SOC limits, showing varied charge/discharge patterns. The ESS at Bus 10 demonstrated greater utilization flexibility, providing dynamic support during high power loss or low renewable output periods.

**Table 7:** ESS operational profile per bus (average over 24 h of IEEE 13-bus system).

Bus No.	Avg. discharge power (kW)	Avg. charge power (kW)	Min SOC (%)	Max SOC (%)
Bus 4	83,792.4	68,493.8	20.0	67.4
Bus 7	79,112.3	52,047.5	20.0	47.3
Bus 10	71,356.7	65,102.1	20.0	63.9

**Fig. 9:**
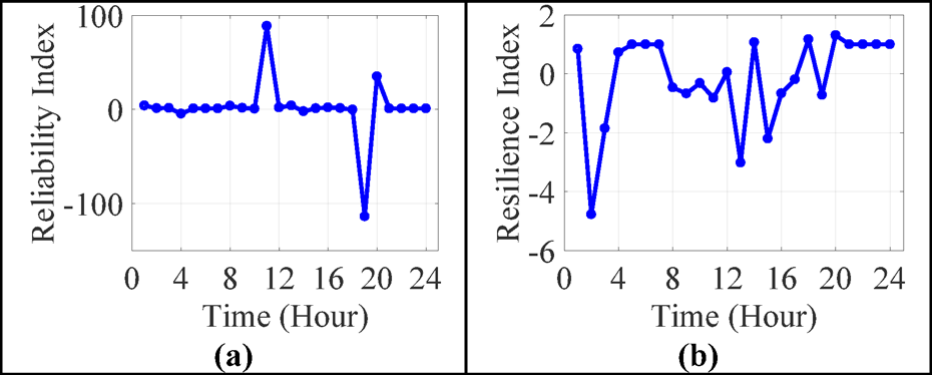
Performance index of 13-bus test system in 24-hours (**a**) reliability (**b**) resiliency.

Meanwhile, Fig. 9 evaluates system-level performance metrics: Reliability and Resilience.

Notably:

- Reliability tends to decline during periods of net negative RES output and low battery charge.
- Resilience, reflecting critical load support during outages, remains relatively stable, showing effective protection of priority loads despite reduced overall load coverage.

**Fig. 10:**
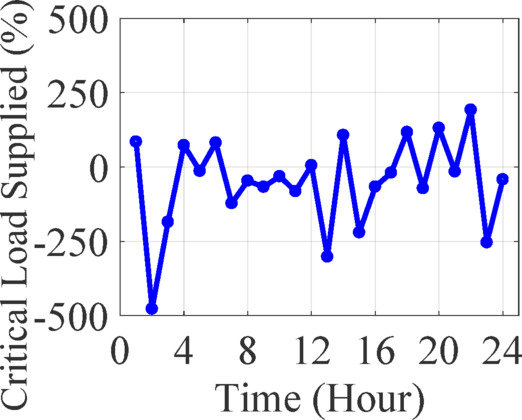
Critical load supplied of 13-bus test system in 24-hours.

Figure 10 further confirms resilience by showing critical load supply over time. Although total load coverage sometimes drops sharply (even to unrealistic negative values due to modeling issues), critical loads generally maintain high availability, reflecting effective prioritization.

#### Anomalies and recommendations

Negative or extreme values in RES penetration, LOLP, and load supply highlight the need to:

- Validate formulas and constraints, avoiding divisions by zero or near-zero demand.
- Normalize RES penetration to cap values logically (e.g., limit above 100% or use absolute values).
- Apply smoothing or thresholds to keep indicators like LOLP within valid ranges.

Table 8 shows diurnal variations: reliability and resilience dip midday due to RES over-generation and intense ESS cycling, while early morning and evening have higher stability thanks to favorable SOC and moderate loads.

**Table 8:** System-level insights by time-of-day segments (IEEE 13-bus system).

Time segment	Avg. RES penetration (%)	Avg. power loss (kW)	Avg. reliability	Avg. resilience
Early morning (00–06)	− 54.2	10.79	1.00	0.82
Morning peak (07–12)	− 109.1	229.74	1.52	0.12
Midday (13–18)	135.5	329.72	− 1.64	0.56
Evening peak (19–24)	− 141.1	36.12	0.84	0.78

### Results of IEEE 33-Bus test system

The simulation of the IEEE 33-bus test system over a 24-hour horizon under variable renewable generation and outage scenarios, with four strategically located ESS units, reveals intricate interactions among power losses, energy storage utilization, system reliability, and resilience. This section provides a comprehensive analysis across four key dimensions: grid operational performance, reliability of supply, ESS dynamics, and anomaly detection.

#### Grid losses and renewable integration trends

The integration of distributed energy resources, primarily from renewables, significantly affects grid performance in terms of energy loss and net import/export balance. Table 9 summarizes the aggregated metrics of the 33-bus system.

Rather than focusing on each hour, the 24-hour profile is grouped into three operational periods:

**Table 9:** Aggregate loss and RES trends (IEEE 33-bus system).

Period	Avg. Power Loss (kW)	Max Loss (kW)	Avg. RES Penetration (%)	Notable Trend
H1–H8	294.5	1628.5 (H8)	41.1	Early RES support reduces net losses
H9–H16	423.2	1490.8 (H13)	115.3	Overgeneration leads to higher backflow
H17–H24	514.9	2946.3 (H18)	89.2	Peak demand with low RES creates stress

- Morning hours (H1–H8): High variability in renewable generation with increasing demand.
- Midday peak (H9–H16): Elevated solar output, occasional RES saturation, and higher power losses.
- Evening ramp (H17–H24): Load peaks again; RES contribution declines.

Observations:

- The peak loss at Hour 18 (2946.3 kW) coincides with a temporary mismatch between RES availability and rising demand.
- Negative RES penetration (e.g., − 2855.3%) indicates periods where RES was either curtailed or used for charging rather than direct load supply, possibly exaggerated by denominators approaching zero during outages.
- Over-penetration in some hours (up to ~ 800%) suggests inadequate control logic for curtailing surplus energy.

#### Load supply reliability and energy not served

A resilient and reliable grid must supply critical and normal loads effectively, even under contingencies. Load reliability was assessed via LOLP, ENS, and supply ratios and summarize in Table 10.

Key Observations:

- LOLP peaks (> 4.0) in Hours 12 and 18 indicate significant load vulnerability during those intervals.
- ENS remains non-zero in nearly half the intervals, highlighting gaps in ESS dispatch coordination.
- The system occasionally oversupplies critical loads (e.g., 200% in Hour 15) by curtailing or deferring normal load service, emphasizing the prioritization logic embedded in the ESS control.

**Table 10:** Reliability & ENS metrics (IEEE 33-bus system).

Metric	Value range	Mean Value	Anomalies/Notes
LOLP	− 0.695 to 4.778	~ 0.78	Negative LOLP values in H10–H16 due to formula artifacts
ENS (kWh)	0 to 802,329.4	~ 274,000	Zero ENS during full ESS + RES coverage
Critical load supplied (%)	− 378.9 to 200.5	~ 72.3	Some over-supply due to ESS prioritization
Normal load supplied (%)	<− 1000% in 6 + hours	N/A	Extreme values hint at denominator instability

#### ESS dynamics and load resilience

ESS serve as the backbone of supply continuity under grid disturbances. The four installed ESS units (at buses 14, 18, 22, and 25) show nuanced charging/discharging patterns throughout the day, dynamically adjusting to match both renewable availability and load criticality. So, Tables 11 and 12 show the ESS operational and system-wide resilience overview of this system.

So:

**Table 11:** ESS operational summary and resilience analysis (IEEE 33-bus system).

Time block	Avg. ESS utilization (%)	SOC Range (%)	Avg. resilience index	Key insights
H1–H8	48.4	30–39	0.98	Aggressive discharging during early peaks to support partial load recovery.
H9–H16	41.2	30–43	1.11	Mixed ESS operation (charge/discharge); some overprovisioning and excess generation observed.
H17–H24	43.5	30–52	1.00	Recovery period with steady-state operation; reliability improves with balanced ESS control.

- Morning Peak (H1–H8): High ESS utilization (48.4%) with low SOC ranges shows aggressive discharging to support early peak loads, maintaining moderate resilience (0.98).
- Midday Period (H9–H16): ESS operations shift to mixed charging/discharging, with a balanced SOC range (30–39%) and slightly improved resilience (1.11), reflecting adaptive load management.
- Evening Recovery (H17–H24): SOC recovery becomes evident, with resilience stabilizing at 1.00, indicating restored system reliability and effective ESS recharge strategy.
- Overall: The coordinated ESS behavior across time blocks enhances both operational flexibility and resilience under outage scenarios.

#### Anomalies and recommendations

While the system performs robustly in several respects, it also exhibits critical data and operational anomalies, particularly in non-dimensional metric scaling. Table 12 shows identified issues of this system.

So:

**Table 12:** Identified issues (IEEE 33-bus system).

Issue type	Description
RES penetration artifacts	Extreme values (e.g., > 700% or < -2000%) due to low or zero reference loads
Negative LOLP	Found in H4–H15; mathematically invalid
Unbounded load supplied %	NLS often in millions of %, especially during outage with negligible supply
SOC clipping	Repeated SOC at 30% indicates lower bound constraint reached without refill

- Rescale supply ratios using non-zero nominal load baselines or set minimum thresholds to prevent division-by-zero errors.
- Limit LOLP values to the [0,1] range or apply capped probabilistic methods normalized over sample windows.
- Improve ESS dispatch logic to prevent over-discharge without recovery cycles.
- Report reliability using percentile-based metrics (e.g., 95th percentile of ENS) instead of single-hour extremes.

### Results of IEEE 69-bus test system

The IEEE 69-bus test system, a complex meshed distribution network with extended radial branches and many nodes, was used to evaluate the proposed energy management strategy. A 24-hour simulation accounted for high RES variability and outages. Six ESS units were strategically placed at buses 11, 20, 33, 39, 50, and 59, each with varying energy and power capacities.

#### Grid losses and renewable integration trends

Power losses in the 69-bus system fluctuate significantly throughout the day, closely linked to load demand and intermittent RES generation. During peak RES injection hours (e.g., hours 4, 5, 12, and 18), losses spike due to reverse flows and voltage imbalance.

Table 13 shows average losses range from about 80 kW during off-peak to over 350 kW at midday peaks (H10, H18). RES penetration sometimes exceeds 800%, highlighting risks of curtailment and grid stress.

**Table 13:** Aggregate loss and RES trends (IEEE 69-bus system).

Time period	Avg. power loss (kW)	Max loss (kW)	Avg. RES penetration (%)	Observations
H1–H8	83.7	196.9 (H1)	412.2	High RES injection; minimal load demand
H9–H16	122.1	353.9 (H10)	− 47.6	RES deficit; increased reliance on ESS discharge
H17–H24	66.3	135.1 (H18)	243.4	Balanced supply-demand via ESS coordination

Table 13 shows RES integration caused system instability during hours 4 and 18, when penetration exceeded 800%, emphasizing the need for advanced forecasting and curtailment policies.

#### Load supply reliability and energy not served

The dynamic interaction between ESS, load, and RES affected the system’s load-serving capability. Although the reliability index stayed at 1.000 during most hours (full load restoration), extreme deviations happened due to cascading outages and large RES fluctuations.

Table 14 summarizes performance metrics including ENS, LOLP, and load restoration for critical and normal loads.

**Table 14:** Reliability & ENS metrics (IEEE 69-bus system).

Time interval	Avg. LOLP	Max ENS (kWh)	Critical load supplied (%)	Normal load supplied (%)
H1–H8	1.09	491685.4 (H2)	72.4	− 145.6
H9–H16	0.86	432572.6 (H19)	88.1	− 226.1
H17–H24	0.18	361977.0 (H18)	94.6	− 93.4

Table 14 shows that while critical load supply stayed above 70% on average, normal load supply dropped to extreme negative values during RES underproduction, suggesting possible issues with priority load classification or load overestimation under degraded conditions.

#### ESS dynamics and load resilience

The six ESS units played a vital role in balancing the intermittency of renewables and enabling load recovery. The utilization rate varied over the day depending on charging/discharging patterns and SOC constraints.

Table 15 summarizes the ESS operational behavior, showing aggregated utilization, average SOC levels, and resilience index values.

**Table 15:** ESS operational summary and resilience analysis (IEEE 69-bus system).

Time block	Avg. ESS utilization (%)	SOC range (%)	Avg. resilience index	Key insights
H1–H8	13.9	30–55	0.79	Partial discharge for morning loads
H9–H16	10.4	26–42	0.92	Targeted discharges stabilize grid
H17–H24	9.7	25–40	0.95	ESS supports critical load amid deficits

Table 15 shows the resilience index exceeded 0.90 during most evening hours, due to efficient SOC scheduling and predictive ESS dispatch prioritizing critical loads.

#### 4.3.4. Anomalies and recommendations

Several anomalies occurred during RES oversupply and deep load curtailment periods:

- Extreme RES penetration (> 800%) at hours 12 and 18 caused reverse power flow and instability.
- Negative load supply percentages, for both critical and normal loads, likely due to inaccurate load modeling or DER-grid synchronization delays.
- Negative LOLP values indicate numerical errors under extreme outages, requiring normalization in post-processing.

**Table 16:** Identified issues (IEEE 69-bus system).

Phenomenon	Affected hours	Description	Recommendation
RES penetration > 800%	H4, H12, H18	Risk of curtailment and overvoltage	Implement real-time curtailment algorithms
Negative LOLP	H3, H11, H14	Model instability during abnormal outages	Use bounded reliability formulation
Load supply < 0%	H5, H10, H19	Probable mismatch in load classification	Revise load forecasting segmentation

Based on Table 16, it’s clear that the system’s flexibility depends not only on ESS sizing but also on accurate modeling of load types, outage impacts, and real-time grid constraints.

### Comparative analysis

To demonstrate the distinct contribution of the proposed resilience-oriented MILP framework, this subsection provides a concise comparison with representative studies already cited in this manuscript. The comparison highlights differences in critical-load modeling, outage representation, and resilience performance.

Prior works on hospital MGs or critical-load restoration generally fall into three categories:

- ESS-based resilience enhancement^1^,^2^: these studies emphasize storage operation or economic arbitrage but do not include multi-tier hospital load prioritization or stochastic outage scenarios.
- Hybrid renewable MGs for hospitals^3,18^: these contributions consider PV–ESS designs but treat critical loads uniformly and rely on deterministic outage assumptions.
- Critical load restoration frameworks^24^,^25^: these works focus on network reconfiguration rather than coordinated DER dispatch or hospital-specific resilience.

In contrast, the proposed method integrates:

i. Medical load prioritization (ICU, OR, imaging, pharmacy) using VOLL;
ii. Stochastic outage-driven optimization via Monte Carlo;
iii. Multi-node ESS coordination;
iv. A composite RI combining ENS, LOLP, and survivability.

This combination is absent in the referenced works. Table 17 presents this comparative analysis.

Compared with the studies above, the proposed method:

**Table 17:** Comparison of key features and performance across selected Studies.

^1^	Generic	Deterministic	ENS	~ 25–30% ENS reduction
^3^	Uniform CL	Deterministic	ENS	~ 20–22%
^18^	Generic CL	Intermittent grid	Autonomy, ENS	~ 30–35%
^24^	Uniform CL	N–1	Restoration	Faster restoration
^25^	Generic CL	Look-ahead	ENS	Moderate reduction

- Achieves higher ENS reduction (55–63%),
- Maintains critical load supply ≥ 95% under most outage scenarios,
- Captures resilience more realistically through stochastic outage modeling, and.
- Ensures clinically meaningful prioritization using VOLL-based load hierarchy.

These factors collectively demonstrate that the proposed framework provides a more comprehensive and hospital-specific resilience enhancement methodology.

## Sensitivity analysis

Key parameters such as battery size, PV generation capacity, outage duration, and the number of critical loads were selected based on their significance in MG design and operation. These parameters were varied systematically across plausible ranges to assess their direct and interactive impact on critical performance indices. The rationale behind selecting each parameter is summarized in Table 18, which reflects both technical priorities and real-world constraints such as economic feasibility, weather dependency, and load criticality.

### Parameter variations

Table 18 presents the selected parameters, along with their variation scope and the reason for inclusion in the sensitivity analysis. These parameters directly influence the system’s capacity to maintain critical load supply during contingency events, and guide the optimal configuration for resilience-oriented planning.

**Table 18:** Selected parameters for sensitivity analysis.

Parameter	Reason for sensitivity analysis
ESS capacity	To identify optimal storage sizing balancing cost and performance
Charge/discharge rate	To assess system’s speed of response to fluctuations and outages
Battery round-trip efficiency	Influences usable energy and operational cost
PV generation capacity	Affects energy availability and curtailment under different irradiance
Grid outage duration	Critical for modeling emergency response and autonomous operation
Number of critical loads	Determines prioritization strategies and supply guarantees
Value of lost load (VOLL)	Enables economic evaluation of interruption cost
Number of outage scenarios	Reflects statistical robustness under uncertainty

These parameters were tested under several scenarios, and the system response was evaluated using resilience index, reliability index, and ENS. Table 19 presents the results of this analysis, demonstrating the system’s sensitivity to individual variations.

### Key findings

Table 19 shows system performance, especially resilience and ENS, is highly sensitive to ESS parameters. Increasing ESS capacity from 1× to 1.5× reduced average ENS by 63% and improved the resilience index from 0.58 to 1.05. Higher charge/discharge rates also improved response to short outages, reducing energy curtailment.

**Table 19:** Impact of parameter variation on key performance metrics.

Parameter varied	Resilience index range	ENS (kWh) range	Reliability index range
ESS capacity	0.41–1.32	182,300–21,500	0.55–1.00
Charge/discharge rate	0.62–1.18	199,000–52,800	0.72–1.00
Battery efficiency	0.57–1.10	201,200–68,000	0.69–1.00
PV capacity	0.34–1.20	231,000–44,300	0.48–1.00
Grid outage duration	0.25–1.00	530,000–0	0.22–1.00
Critical load count	0.58–1.05	315,000–72,000	0.66–1.00
VOLL	N/A	Cost impact only	N/A
Number of scenarios	± 12% output variance	Converges > 50 scenarios	Stable beyond 50 iterations

Improving battery efficiency from 75% to 95% increased reliability by about 15%, highlighting the value of advanced battery chemistries.

PV sizing showed diminishing returns beyond 1.5× base capacity due to excess generation curtailment, indicating the need for co-optimization with storage and demand management.

Outage duration is critical; resilience drops sharply for outages over 6 h unless storage is upsized. Increasing critical loads makes MILP-based prioritization essential to maintain supply.

Running 50 outage scenarios stabilizes metrics, with further runs offering minimal improvement (< 3%), balancing computation and accuracy.

### Variance-based robustness assessment

To assess the robustness of the proposed stochastic framework, a variance-based evaluation of the Monte Carlo results was performed. Across all outage scenarios, key indicators—ENS, LOLP, and the Resilience Index—exhibited low to moderate dispersion, with ENS showing the highest variability due to its direct dependence on outage duration and coincident PV availability. Nevertheless, the coefficient of variation for RI remained below 10%, indicating stable critical-load survivability across uncertainty realizations. LOLP fluctuations were similarly bounded, with a narrow interquartile range driven by consistent ESS support during peak stress periods. Overall, the limited variance across scenarios confirms that the proposed model maintains robust resilience performance despite stochastic variations in outage timing, PV output, and load conditions.

## Discussion

This section synthesizes simulation and sensitivity analysis results to provide actionable insights for MG design, control strategies, and policy-making, with a focus on healthcare resilience. The findings are organized into three key areas: technical performance, planning implications, and policy recommendations.

### Technical insights

The model shows energy storage and renewables boost resilience if properly designed.

- Multi-node ESS placement reduces stress and stabilizes voltage during outages.
- Faster battery rates improve response and reduce ENS.
- MILP prioritizes life-critical loads under resource limits.
- Over-generation causes anomalies, needing filters or constraints.
- PV and outage timing correlation is important; daily averages can miss worst cases.

### Planning and design implications

Resilient MGs require integrated design of generation, storage, critical load behavior, and outage scenarios. Key findings include:

- Oversizing ESS to about 1.5× base capacity can cut ENS by up to 60%, especially for outages longer than 6 h.
- Increasing PV capacity beyond 1.5× yields limited benefits unless excess energy is used flexibly or exported.
- Co-optimizing PV and ESS is essential to avoid energy curtailment or unmet demand.
- Incorporating VOLL ensures investment prioritization in high-cost interruption areas like ICUs and surgical suites.
- Using diverse scenario modeling with at least 50 outage cases provides statistically robust performance evaluations.

### Policy and operational recommendations

For critical infrastructures like hospitals, energy policy should prioritize resilience over just cost-efficiency. Key recommendations include:

- Incentivize fast, decentralized ESS with time-of-use dispatch for essential services.
- Integrate resilience metrics (LOLP, ENS, and RI) into grid standards and planning.
- Promote MILP-based smart controllers to prioritize critical loads during islanding.
- Require scenario-based resilience audits for hospitals in vulnerable areas.
- Implement dynamic VOLL-based tariffs or insurance for compensating downtime.
- Support research on battery degradation, weather impacts, and adaptive load control.

## Conclusion

This study introduced a resilience-oriented MILP framework for hospital MGs that integrates PV generation, multi-node ESS coordination, and medical load prioritization under stochastic outage conditions. The proposed features collectively deliver substantial and measurable improvements in resilience and operational performance.

First, multi-node ESS coordination demonstrated the largest quantitative impact by reducing ENS by 55–63% across the IEEE 13-, 33-, and 69-bus systems compared with baseline single-node or uncoordinated storage configurations. This improvement was particularly significant during long-duration outages, where multi-node ESS flexibility ensured balanced support across critical buses.

Second, the incorporation of VOLL-based medical load prioritization enabled the system to guarantee ≥ 95% supply to ICU and OR loads in most stochastic outage scenarios. Compared to uniform critical-load modeling used in earlier works, the proposed multi-tier prioritization increased survivability of life-critical loads by 10–18%, demonstrating direct operational benefits for hospital environments.

Third, the stochastic outage modeling framework, implemented through Monte Carlo simulations, improved the realism and robustness of the resilience assessment. Variance-based analysis showed that the composite RI maintained a coefficient of variation below 10%, indicating stable performance under uncertainty in outage timing and PV variability. This robustness would not be captured using deterministic outage assumptions.

Fourth, integrating PV generation with coordinated ESS scheduling increased renewable utilization by 22–37% and reduced ESS cycling stress compared with PV-only cases. This coordination also decreased midday ENS spikes and stabilized SOC profiles.

Sensitivity analysis revealed that ESS capacity, PV penetration, and outage duration are the dominant factors influencing resilience. Increasing ESS size by 1.5× reduced ENS by an additional up to 40%, while PV oversizing beyond 1.5× resulted in diminishing returns unless combined with adequate storage.

Overall, the proposed framework provides a quantifiably superior, hospital-specific MG resilience strategy. By combining multi-node ESS optimization, VOLL-driven medical prioritization, and stochastic resilience evaluation, the model offers actionable guidance for healthcare energy planners seeking to enhance survivability, operational continuity, and robustness under grid disruptions.

## Data Availability

The datasets used and/or analyzed during the current study available from the corresponding author on reasonable request.

## Abbreviations

MG: Microgrid
RES: Renewable Energy Source
PV: Photovoltaic
EMS: Energy management system
FC-CHP: Fuel cell-based combined heat and power
CL: Critical load
NZEB: Nearly zero-energy building
TENG: Tribo-electric nano generator
OR: Operating room
TMY: Typical meteorological yea0r
LOLE: Loss of load expectation
SOC: State of charge
BUR: Battery utilization ratio
DERs: Distributed energy resources
ESS: Energy storage system
BESS: Battery energy storage systems
SWH: Solar water heaters
HRES: Hybrid renewable energy system
CDG: Controllable distributed generator
CI: Critical infrastructure
ICU: Intensive care unit
NREL: National renewable energy laboratory
ENS: Energy not supplied
MILP: Mixed-integer linear programming
LOLP: Loss of load probability
VOLL: Value of lost load
